# Validation of the Italian Version of the Functional Oral Intake Scale (FOIS-It) Against Fiberoptic Endoscopic Evaluation of Swallowing and Nutritional Status

**DOI:** 10.1007/s00455-021-10257-9

**Published:** 2021-02-16

**Authors:** Aurora Ninfa, Nicole Pizzorni, Angelo Eplite, Claudia Moltisanti, Antonio Schindler

**Affiliations:** 1 Phoniatric Unit, Department of Biomedical and Clinical Sciences “Luigi Sacco”, University of Milan, Via GB Grassi 74, 20154 Milan, Italy; 2 Department of Pathophysiology and Transplantation, University of Milan, Via Francesco Sforza 35, 20122 Milan, Italy

**Keywords:** Deglutition, Deglutition disorders, Functional oral intake, Validation, Fiberoptic endoscopic evaluation of swallowing, Nutritional status

## Abstract

The Functional Oral Intake Scale (FOIS) is a reliable and valid tool to assess functional oral intake of food and liquids in patients with oropharyngeal dysphagia (OD). Its validity was established for stroke patients against Videofluoroscopic Swallowing Study in English and Chinese and against Fiberoptic Endoscopic Evaluation of Swallowing (FEES) in German. FOIS was cross-culturally validated into Italian (FOIS-It), but construct validity against instrumental assessment and nutritional status was not investigated. The study aims at contributing to the validation of the FOIS-It, by performing convergent and known-group validity against FEES and nutritional status in patients with OD of different etiologies. Overall, 220 adult patients with OD of etiological heterogeneity were recruited. FOIS-It score and Body Mass Index (BMI) were collected. FEES was performed to assess swallowing safety and efficiency based on the Penetration-Aspiration Scale (PAS) and the Yale Pharyngeal Residue Severity Rating Scale (YPRSRS). Moderate to weak associations with PAS (*ρ* = − .37, *p* < .01), YPRSRS in the pyriform sinuses (*ρ* = − .20, *p* < .01), and BMI (*ρ* = .24, *p* < .01) were detected with Spearman’s correlation. FOIS-It distribution was compared with the Mann–Whitney U and Kruskal–Wallis tests. Significantly lower FOIS-It scores were detected among patients with penetration/aspiration (PAS > 2) and penetration (PAS > 2 ≤ 5) for all consistencies (*p* < .01), aspiration (PAS > 5) of liquids and semisolids (*p* < .001), residue in the pyriform sinuses (YPRSRS > 3) with semisolids (*p* < .001) and solids (*p* = .02), and malnutrition (BMI ≤ 18.5; *p* = .019). FOIS-It appears as a valid tool to assess functional oral intake against FEES’ measures of swallowing safety and efficiency and nutritional status in patients with OD of etiological heterogeneity.

## Introduction

Oropharyngeal Dysphagia (OD) is a multifactorial clinical condition defined as any abnormality in oropharyngeal swallowing physiology resulting from any disease that leads to impaired swallowing safety and/or efficiency [1, 2]. Swallowing safety and swallowing efficiency represent the ability to transfer the bolus from the mouth to the stomach without penetration or aspiration into the lower airways and without post-swallowing pharyngeal residue, respectively. If impaired, respiratory complications (i.e. aspiration pneumonia) and nutritional compromise may arise [2]. Videofluoroscopic Swallowing Study (VFSS) and Fiberoptic Endoscopic Evaluation of Swallowing (FEES) are a recognized gold standard for the instrumental assessment of swallowing safety and efficiency [3].

In everyday clinical practice, modifications of the oral intake type are adopted as compensatory strategies (e.g. food modifications) and alternative feeding methods (e.g. nasogastric (NG-tube) or gastrostomy (G-tube) tube) [2, 4, 5]. Nevertheless, hospitalized and institutionalized individuals on texture-modified regimens are reported as having a reduced oral intake, with suboptimal fluid, energy, and protein consumption [6, 7], as well as macro and micro-nutrients deficits [8].

The Functional Oral Intake Scale (FOIS) is a reliable tool for the daily level assessment of the functional oral intake of food and liquids in patients with OD. It is widely used for clinical and research purposes in different patient populations with OD as it is easy to apply and provides a communal and standardized language. FOIS was originally developed for English-speaking populations [9] and subsequently validated in Chinese [10], Italian [11], and German [12].

Previous studies established inter-rater reliability, responsiveness, and construct validity, reporting overall consistent results. According to the COnsensus‐based Standards for the selection of health Measurement INstruments (COSMIN) [13], *construct validity* is defined as the degree to which the scores of an instrument are consistent with the hypotheses concerning internal relationships, relationships with scores of other instruments *(convergent validity)*, or differences between relevant groups *(known-group validity)* [13]. In particular, a satisfactory convergent validity is an index of the capacity of an instrument to adequately reflect relationships between the construct assessed and others theoretically related. A satisfactory known-group validity, instead, proves the capacity of an instrument to reflect, through its scores, differences in target groups, which are theoretically expected to differ in the construct to be measured.

Considering FOIS construct validity, it is worth noting that FEES was applied for the instrumental assessment of swallowing safety and efficiency only in the German version of the scale, while VFSS was used in the original and the Chinese validations. Moreover, previous FOIS validations were carried out uniquely on stroke patients, except for the Italian one which involved individuals with dysphagia due to different etiologies recruited from various healthcare settings by speech and language pathologists (SLPs) with a wide range of clinical experience. However, the Italian validation focused only on cross-cultural adaptation following the 5-stage process described by Beaton et al. [14] and subsequent testing of inter-rater reliability, and face validity against an ad hoc developed questionnaire which contained information similar to FOIS-It. Thus it did not explore the possible relationship between functional oral intake and swallowing safety and efficiency. To date, FOIS is used within different healthcare settings and for the assessment of patients with a wide range of diseases [15–17]. Patients with heterogeneous clinical conditions may differ in pathological swallowing patterns as well as in coping with swallowing symptoms since they adapt to chronic disorders or may be recovering from an acute event. As a result, diverse association patterns between functional oral intake and swallowing safety and efficiency may be revealed addressing a heterogeneous OD population. Therefore, extending the FOIS validation to clinical conditions other than stroke appears as a pivotal step.

Crary et al. [9] suggested broadening FOIS validation addressing the consequences of reduced functional oral intake on nutritional status. OD is reported as being an independent risk factor for malnutrition [18]. The European Society of Clinical Nutrition and Metabolism (ESPEN) guidelines define malnutrition as a state resulting from the lack of intake or uptake of nutrition, due to starvation, disease or advanced aging (e.g. > 80 years), alone or in combination, which lead to altered body composition (decreased fat-free mass) and body cell mass contributing to diminished physical and mental function and impaired clinical outcome [19]. Among the several conditions encompassed under this umbrella term, disease-related malnutrition without inflammation acknowledges a reduced nutritional intake associated with OD as an etiologic mechanism [19].

The present study aims at contributing to the validation of the Italian version of the FOIS (FOIS-It), performing convergent and known-group validity against FEES and nutritional status in adult patients with OD from etiological heterogeneity. In particular the FOIS-It scores were examined in order to test potential correlations with swallowing safety and efficiency and nutritional status and to distinguish between (i) patients with and without penetration/aspiration, and residue on FEES and, (ii) patients with normal nutritional status and malnourished patients. Moderate inverse correlations were expected between FOIS-It scores and swallowing safety and efficiency measured with the Penetration Aspiration Scale (PAS) and the Yale Pharyngeal Residue Severity Rating Scale (YPRSRS), respectively. Direct correlations were expected between FOIS-It and nutritional status expressed as BMI. Patients who presented penetration/aspiration, penetration, aspiration, or residue on FEES and malnutrition based on BMI, compared to those without, were hypothesized as scoring significantly lower at FOIS-It.

## Material and Methods

### Study Design

This cross-sectional study was carried out according to the Declaration of Helsinki and was previously approved by the Institutional Review Board of the Luigi Sacco Hospital. All participants provided written informed consent. Data concerning functional oral intake, swallowing safety and efficiency and nutritional status were collected prospectively for ongoing research studies pertaining to swallowing function and nutritional status in patients with OD. For the purposes of the present study, secondary analyses were retrospectively performed on the collected data.

### Subjects

Participants were selected among the consecutive cohort of patients who had been referred to the Phoniatric clinic in the Luigi Sacco Hospital (Milan, Italy) for known or suspected OD in order to undergo a FEES examination between 2017 and 2020. Only patients who were participating in ongoing research studies on swallowing performance were considered eligible for the present study, as they had undergone the same FEES protocol and had given their informed consent. Participants who had undergone FEES for known or suspected OD, and /or reported information about functional oral intake met the inclusion criteria and were selected for the study. Pediatric patients (age < 18 years) were excluded from the study.

### Data Collection

Demographic, clinical, and instrumental data were recorded and stored anonymously on the system’s hard drive and saved on external memory drives as backup copies. Prior to FEES, patients' typical oral intake was recorded following the Italian version of the Functional Oral Intake Scale (FOIS-It) [9, 11]. Height and weight were measured. Subsequently, the FEES examination was performed using a XION EF-N flexible endoscope with a diameter of 3.4 mm and a length of 320 mm (XION GmbH, Berlin, Germany) mounted on an EndoSTROBE camera (XION GmbH, Berlin, Germany). The same experienced Phoniatrician carried out all the FEES examinations. They were conducted with liquids (5–10–20 ml of blue-dyed water × 3 trials for each volume; International Dysphagia Diet Standardisation Initiative [20]—IDDSI 0; < 50 mPa s at 50 s^−1^ and 300 s^−1^), semisolids (5–10–20 ml of pudding × 3 trials for each volume; IDDSI 4; 2583.3 ± 10.41 mPa s at 50 s^−1^ and 697.87 ± 7.84 mPa s at 300 s^−1^), and solids (half biscuit × 2 trials; IDDSI 7 Regular). The bolus administration order was the same for all the patients, starting with liquids (3 × 5 ml, 3 × 10 ml, 3 × 20 ml), followed by semisolids (3 × 5 ml, 3 × 10 ml, 3 × 20 ml), and finally solids. The protocol was reduced in case either consistency or volume was not considered safe for administration or if severe swallowing efficiency impairment was observed. All the examinations were stored in an anonymous form in.AVI format.

### Outcome Measures

#### Functional Oral Intake

The FOIS-It [9] is a 7-point ordinal scale describing the functional level of oral intake of food and liquids. Level 7 represents a full oral diet with no restrictions, levels 6–4 indicate a full oral diet with restrictions, levels 3–2 describe a mixed oral and tube intake, while level 1 represents a totally tube-dependent intake.

#### Swallowing Safety and Swallowing Efficiency

Swallowing safety and efficiency were investigated based on FEES video-recordings. Each FEES was assessed by the same Phoniatrician with > 20 years of clinical and research experience in dysphagia.

Swallowing safety was assessed using the Penetration-aspiration scale (PAS) [21, 22]. The ordinal scale scores from 1 to 8, with score 1 representing no penetration and aspiration, score 2 representing transient penetration with ejection, scores 3 to 5 laryngeal penetration without ejection and/or reaching the vocal folds, and scores 6 to 8 tracheal aspiration. As recently reported in studies assessing PAS psychometric properties [23, 24], PAS scores 1–2 were considered to reflect normal swallowing function. Penetration/aspiration, penetration, and aspiration were scored as present with PAS > 2 [23, 24], PAS > 2 ≤ 5, and PAS > 5 [21], respectively.

As a measure of swallowing efficiency, pharyngeal residue was rated according to the Yale Pharyngeal Residue Severity Rating Scale (YPRSRS) [25]. This ordinal scale provides two scores based on the amount of post-swallow residue in the valleculae and the pyriform sinuses. The score ranges from 1 (no residue) to 5 (severe residue). For the present study, as recently reported in the literature [26], a YPRSRS score > 3 was considered suggestive of clinically relevant residue, since coatings (YPRSRS = 2) were commonly reported in a healthy adult population [27].

The worst PAS and YPRSRS scores for each consistency and for each parameter of swallowing safety and efficiency were considered for the analysis.

#### Nutritional Status

Within the clinical assessment, patient’s weight and height were recorded and BMI was calculated (dividing weight in kilograms by height in meters squared) as a measure of nutritional status. According to the international literature, a BMI score ≤ 18.5 is considered suggestive of a condition of malnutrition [19].

### Statistical Analysis

Data are reported as absolute (relative) frequencies. By reason of its ordinal nature, FOIS-It data are reported as median (interquartile range—IQR).

To perform convergent validity, due to the ordinal nature of the outcome variables, correlation analyses between FOIS-It and PAS, YPRSRS, and BMI were performed using Spearman’s test. Correlations were considered strong for *ρ* > 0.5, moderate for values of *ρ* ranging between 0.3 and 0.5, and weak for *ρ* < 0.3 [28]. Driven by the suggestions in the recent literature [23, 24] to treat PAS as a categorical scale and in accordance with the aim of the present study to perform known-group validity of the FOIS-It, the measures of swallowing safety and efficiency for each consistency, and nutritional status were dichotomized. Moreover, converting these measures into categorical ratings led to the verification of FOIS-It scores reflecting clinically relevant differences regarding swallowing safety and efficiency, and malnutrition. FOIS-It scores were compared between the dichotomized groups for swallowing safety (PAS > 2 for penetration/aspiration, PAS > 2 ≤ 5 for penetration, and PAS > 5 for aspiration), swallowing efficiency (YPRSRS > 3), and nutritional status (BMI ≤ 18.5) using the non-parametrical Mann–Whitney U and Kruskal–Wallis tests. Mann–Whitney U test was used for post hoc analysis in case a significant difference at Kruskal–Wallis test was found. A *p*-value smaller than 0.05 was considered significant. In case of multiple comparison, Bonferroni’s correction was applied. All the statistical procedures were carried out with IBM SPSS Statistics 26.0® package for Mac (SPSS Inc, Chicago, IL). The software R 3.6.3 version for Mac (R Core Team (2020). R: A language and environment for statistical computing. R Foundation for Statistical Computing, Vienna, Austria. URL https://www.R-project.org) was used to produce the scatterplots for the graphical representation of the convergent validity analyses. Missing values were excluded from the analysis.

## Results

### Subjects

Based on the inclusion criteria, 220 patients with OD were recruited for the present study. The demographic and clinical characteristics of the sample are shown in Table 1.

**Table 1:** Demographic and clinical characteristics of the recruited patients

Variable	Mean ± SD or *n* (%)
Age	
Years	65.2 ± 13.5
Gender	
M	120 (54.5)
F	100 (45.5)
Diagnosis	
Huntington disease	59 (26.8)
Amyotrophic lateral sclerosis	48 (21.8)
Parkinson’s disease/Parkinsonism	29 (13.2)
Stroke	23 (10.5)
Head and neck cancer	18 (8.2)
Multiple system atrophy	9 (4.1)
Steinert myotonic dystrophy	6 (2.7)
Brain tumor	4 (1.8)
Others^a^	24 (10.9)

^a^Alzheimer’s Disease; Aneurysm; Obstructive Chronic Bronchopneumopathy; Corticobasal Degeneration; Dermatomyositis; Zenker’s Diverticulum; Giullan-Barré Syndrome; Cardiac surgery; Cerebral hypoxia; Gastric Tumor; Systemic Sclerosis; Myopathy; Extrapyramidal Disorder; Syringomyelia; Traumatic Brain Injury; Cerebral Vasculitis

**Fig. 1:**
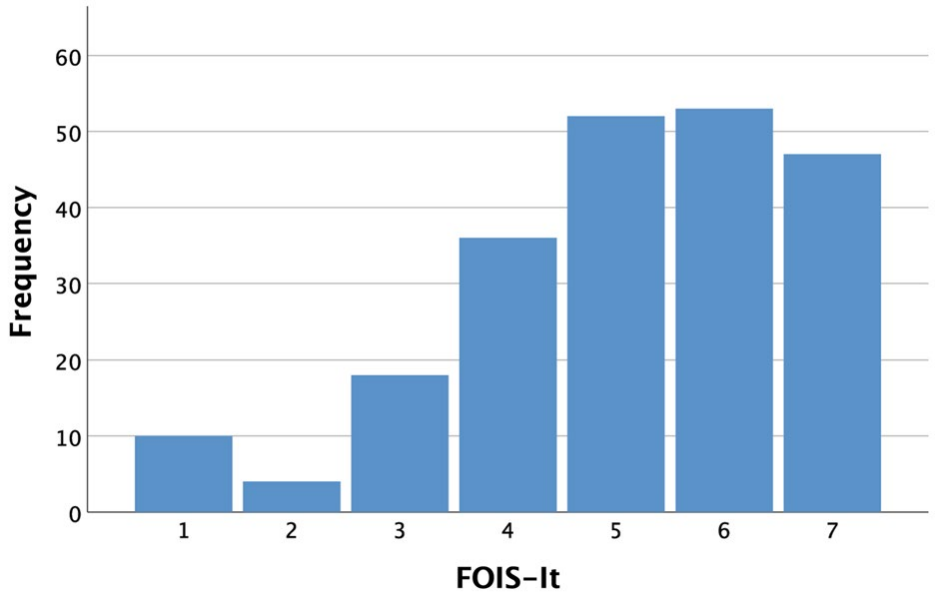
FOIS-It distribution

Figure 1 shows FOIS-It distribution. FOIS-It median value was 5.00 (IQR 4.00–6.00); all the FOIS-It levels were represented, with 32 (14.5%) tube-dependent patients (FOIS-It 1–3), either with NG-tube or G-tube, and 188 (85.5%) patients with full-per-oral nutrition (FOIS-It 4–7). Of these, 36 (16.4%) participants had a total oral intake of a single consistency (FOIS-It 4); 105 (47.7%) had a total oral intake of multiple consistencies requiring special preparation (FOIS-It 5) or without special preparation, but avoiding specific foods or liquid items (FOIS-It 6); while 47 (21.4%) had a total oral intake without texture modifications (FOIS-It 7).

FEES protocol was reduced in case consistency or volume was not considered safe to be administered or if severe swallowing efficiency impairment was observed. In particular, one patient was not tested for liquid boluses because of laryngospasm during the water test in the clinical assessment. Semisolids were not tested on 3 occasions due to severe impairment in the oral control of the bolus. Solids where not tested in 52 patients who were not able to prepare the bolus because of severe impairment of the oral preparatory swallowing stage. Weight and/or height were not measured in 16 occasions resulting in 16 missing BMI scores.

### FOIS-It and Swallowing Safety

**Fig. 2:**
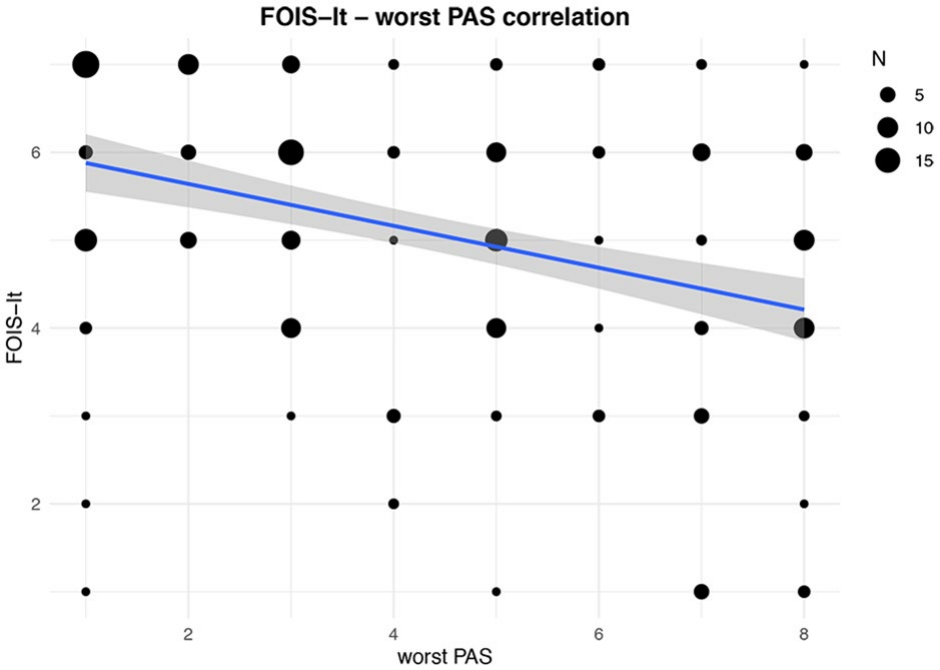
Correlation between FOIS-It and PAS worst score. The line of best fit with 95% confidence interval is displayed. The legend reports the number of patients corresponding to each dot dimension

Penetration/aspiration (PAS > 2) with liquids, semisolids and solids was found in 152 (69.1%), 75 (34.1%) and 22 (10.0%) patients, respectively. Penetration (PAS > 2 ≤ 5) with liquids, semisolids and solids was detected in 86 (39.1%), 58 (26.4%), and 17 (7.7%) patients, while aspiration (PAS > 5) of liquids, semisolids and solids was found in 66 (30.0%), 17 (7.7%) and 5 (2.3%) patients, respectively. Concerning convergent validity, as reported in Fig. 2, a moderate inverse correlation between the FOIS-It and the PAS worst score (*ρ* = − 0.37, *p* < 0.01) was found using Spearman’s test. As shown in Table 2, when considering the different consistencies separately, weak to moderate inverse correlations were detected between the FOIS-It and PAS. Addressing known-group validity, patients with penetration/aspiration for either liquids, semisolids, and solids and with an aspiration of liquids and semisolids scored significantly lower (*p* < 0.05) at FOIS-It compared to patients with a safer swallowing function. Significant differences in FOIS-It distribution were not detected comparing patients with and without aspiration of solids. Moreover, FOIS-It distribution was found significantly different (*p* < 0.05) when comparing patients with different levels of swallowing safety (PAS ≤ 2, PAS > 2 ≤ 5, PAS > 5) with all consistencies. Post hoc analyses highlighted that pairwise comparisons were significant for liquids (PAS ≤ 2 vs PAS > 2 ≤ 5, *p* < 0.01; PAS ≤ 2 vs PAS > 5, *p* < 0.01), semisolids (PAS ≤ 2 vs PAS > 5, *p* < 0.01; PAS > 2 ≤ 5 vs PAS > 5, *p* = 0.01) and for the worst PAS score among all consistencies (PAS ≤ 2 vs PAS > 2 ≤ 5, *p* < 0.01; PAS ≤ 2 vs PAS > 5, *p* < 0.01; PAS > 2 ≤ 5 vs PAS > 5, *p* = 0.02). When dividing patients for swallowing safety with a solid consistency, FOIS-It distribution significantly differed only comparing patients with PAS ≤ 2 and PAS > 2 ≤ 5 (*p* < 0.01). In the comparisons between PAS > 2 ≤ 5 vs PAS > 5 groups with liquids (*p* = 0.06) and the worst PAS score (*p* = 0.06), PAS ≤ 2 vs PAS > 2 ≤ 5 groups with semisolids (*p* = 0.07), and PAS ≤ 2 vs PAS > 5 groups with solids (*p* = 0.10) a trend was found. The results are shown in Table 3.

**Table 2:** Spearman’s correlation coefficients (ρ) between FOIS-It and PAS and YPRSRS in the Valleculae and Pyriform sinuses

Consistency	PAS	YPRSRS valleculae	YPRSRS pyriform sinuses
Liquids	− .35**	.017	− .04
Semisolids	− .23**	− .14*	− .24**
Solids	− .26**	− .09	− .27**
Worst score	− .37**	− .10	− .20**

*p < .05; **p < .01

**Table 3:** Results of the Mann–Whitney U and Kruskal–Wallis tests for the comparison of FOIS-It between patients with and without penetration/aspiration (PAS > 2), penetration (PAS > 2 ≤ 5), and aspiration (PAS > 5) on FEES

Bolus type	Sign of dysphagia	FOIS-It			
		N	Median (IQR)	Mann–Whitney U/Kruskal–Wallis test	*p* value
Liquids	Penetration/aspiration				
	PAS ≤ 2	67	6.0 (5.0–7.0)	3193.000	** < .001**
	PAS > 2	152	5.0 (4.0–6.0)		
	Penetration				
	PAS ≤ 2	67	6.0 (5.0–7.0)	25.477	** < .001**
	PAS > 2 ≤ 5	86	5.0 (4.0–6.0)		
	PAS > 5	66	4.5 (3.0–6.0)		
	Aspiration				
	PAS ≤ 5	153	6.0 (5.0–7.0)	3350.500	** < .001**
	PAS > 5	66	4.5 (3.0–6.0)		
Semisolids	Penetration/aspiration				
	PAS ≤ 2	142	6.0 (5.0–7.0)	3798.000	** < .001**
	PAS > 2	75	5.0 (4.0–6.0)		
	Penetration				
	PAS ≤ 2	142	6.0 (5.0–7.0)	18.784	** < .001**
	PAS > 2 ≤ 5	58	5.0 (4.0–6.0)		
	PAS > 5	17	4.0 (2.0–5.0)		
	Aspiration				
	PAS ≤ 5	200	5.0 (4.0–6.0)	799.500	** < .001**
	PAS > 5	17	4.0 (2.0–5.0)		
Solids	Penetration/aspiration				
	PAS ≤ 2	146	6.0 (5.0–7.0)	871.500	** < .001**
	PAS > 2	22	4.5 (3.0–6.0)		
	Penetration				
	PAS ≤ 2	146	6.0 (5.0–7.0)	12.913	**.002**
	PAS > 2 ≤ 5	17	5.0 (3.5–6.0)		
	PAS > 5	5	4.0 (1.0–6.0)		
	Aspiration				
	PAS ≤ 5	163	6.0 (5.0–7.0)	205.000	.051
	PAS > 5	5	4.0 (1.0–6.0)		
Worst score	Penetration/aspiration				
	PAS ≤ 2	61	6.0 (5.0–7.0)	2810.000	** < .001**
	PAS > 2	158	5.0 (4.0–6.0)		
	Penetration				
	PAS ≤ 2	61	6.0 (5.0–7.0)	29.309	** < .001**
	PAS > 2 ≤ 5	89	5.0 (4.0–6.0)		
	PAS > 5	69	5.0 (3.0–6.0)		
	Aspiration				
	PAS ≤ 5	150	6.0 (5.0–7.0)	3393.500	** < .001**
	PAS > 5	69	5.0 (3.0–6.0)		

Statistically significant *p* are reported in bold

### FOIS-It and Swallowing Efficiency

**Fig. 3:**
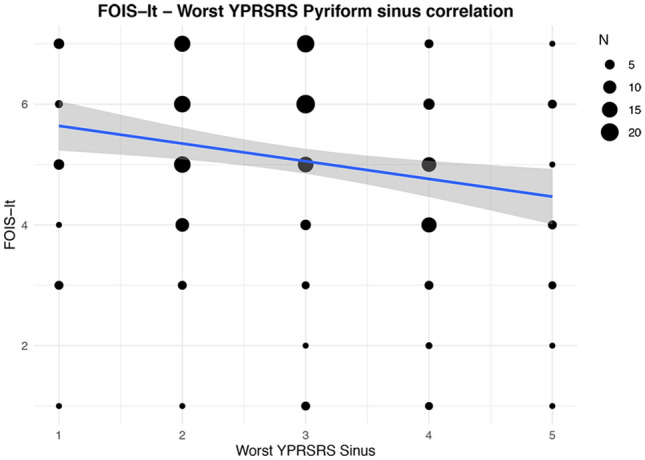
Correlation between FOIS-It and YPRSRS Pyriform sinus worst score. The line of best fit with 95% confidence interval is displayed. The legend reports the number of patients corresponding to each dot dimension

**Fig. 4:**
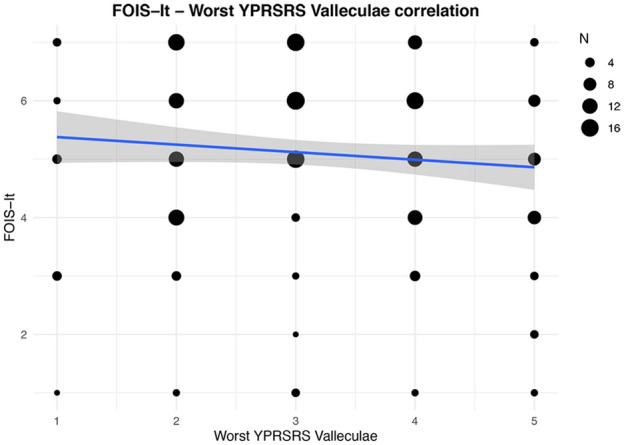
Correlation between FOIS-It and YPRSRS Valleculae worst score. The line of best fit with 95% confidence interval is displayed. The legend reports the number of patients corresponding to each dot dimension

Considering swallowing efficiency, a residue (YPRSRS > 3) in the valleculae with liquids, semisolids and solids was detected in 11 (5.0%), 69 (31.4%) and 44 (20.0%) patients, respectively. A residue in the pyriform sinuses (YPRSRS > 3) with liquids, semisolids and solids was detected in 22 (10.0%), 47 (21.4%) and 14 (6.4%) patients, respectively. As to convergent validity, using Spearman’s test a weak inverse correlation between the FOIS-It and the YPRSRS worst score for the pyriform sinuses (*ρ* = − 0.20, *p* < 0.01) was detected (Fig. 3). No significant correlation between the FOIS-It and the YPRSRS worst score for the valleculae (Fig. 4) was found. As shown in Table 2, the analyses on the single consistencies showed weak inverse correlations between FOIS-It and residue in the pyriform sinuses, with the exception of liquids, and a weak correlation between the FOIS-It and residue in the valleculae only with semisolids. Concerning known-group validity, significantly lower FOIS-It scores (*p* < 0.05) were found in patients who presented residue in the valleculae with semisolids, in the pyriform sinuses with semisolids and solids, and with the YPRSRS worst score for the valleculae and pyriform sinuses. For all the consistencies tested, no significant differences in FOIS-It distribution were detected comparing patients with and without residue in the valleculae (Table 4).

**Table 4:** Results of the Mann–Whitney U test for the comparison of FOIS-It between patients with and without residue (YPRSRS > 3) on FEES

Bolus type	Sign of dysphagia	FOIS-It			
		*N*	Median (IQR)	Mann–Whitney U test	*p* value
Liquids	Residue valleculae				
	Present	11	5.0 (4.0–6.0)	963.500	.368
	Absent	208	5.0 (4.0–6.0)		
	Residue pyriform sinus				
	Present	22	5.0 (4.0–6.0)	1899.500	.332
	Absent	197	5.0 (4.0–6.0)		
Semisolids	Residue valleculae				
	Present	69	5.0 (4.0–6.0)	4171.000	**.027**
	Absent	148	5.0 (4.25–6.0)		
	Residue pyriform sinus				
	Present	47	4.0 (3.0–6.0)	2486.000	** < .001**
	Absent	170	6.0 (5.0–6.25)		
Solids	Residue valleculae				
	Present	44	5.0 (5.0–6.0)	2329.500	.137
	Absent	124	6.0 (5.0–7.0)		
	Residue pyriform sinus				
	Present	14	5.0 (4.0–6.0)	690.500	**.021**
	Absent	154	6.0 (5.0–7.0)		
Worst score	Residue valleculae				
	Present	90	5.0 (4.0–6.0)	4841.000	**.033**
	Absent	129	5.0 (4.0–7.0)		
	Residue pyriform sinus				
	Present	62	4.0 (4.0–6.0)	3102.500	** < .001**
	Absent	157	6.0 (5.0–7.0)		

Statistically significant *p* are reported in bold

### FOIS-It and Nutritional Status

**Fig. 5:**
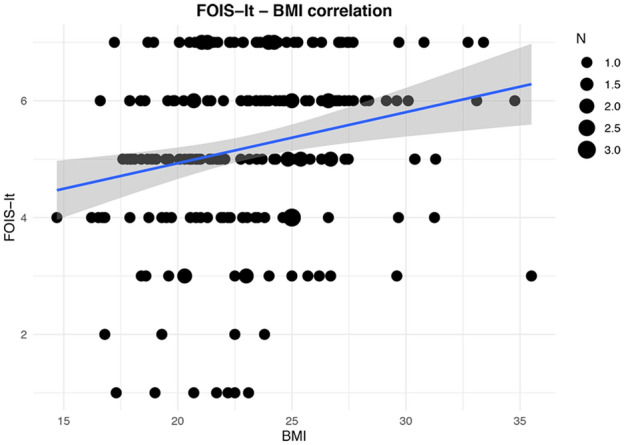
Correlation between FOIS-It and BMI. The line of best fit with 95% confidence interval is displayed. The legend reports the number of patients corresponding to each dot dimension

Twenty (9.8%) patients presented malnutrition, with a BMI ≤ 18.5. Testing convergent validity, a weak direct correlation between the FOIS-It and BMI (*ρ* = 0.24, *p* < 0.01) scores was found (Fig. 5). As regards known-group validity, malnourished patients reported significantly lower FOIS-It scores (median 5.0, IQR 4.0–5.75) compared to patients with normal nutritional status (median 6.0, IQR 4.0–6.5; Mann–Whitney U test = 1273.500, z-score = − 2.342, *p* = 0.019).

## Discussion

The aim of the present study was to contribute to the construct validity of the Italian version of the FOIS against FEES and nutritional status by performing convergent and known-group validity in adult patients with OD of etiological heterogeneity. Consistent with our hypothesis, overall FOIS-It is confirmed as a valid tool to assess functional oral intake.

FOIS validity had previously been established in stroke populations [9, 10, 12]. With the present study, FOIS-It validation has been extended to patients with OD of etiological heterogeneity. Notably the participants in the present study were pooled from different consecutive cohorts of patients participating in ongoing research projects on swallowing performance. One of these focused on patients affected by neurodegenerative diseases which explained why the sample included in the present study was heavily weighted with these etiologies. All FOIS-It levels were represented within the recruited sample, highlighting adequate applicability of the scale with OD from conditions other than stroke. Crary et al. [9] called attention to the lack of scales to specifically assess, with sound psychometric properties, the functional oral intake of food and liquids in patients with OD deriving from any health condition. Thus, the results of the present study appear clinically relevant as they contribute to achieving a communal and standardized language to document the functional oral intake in patients with OD. The sample recruited for the present study amounted to 220 participants. English, Chinese, and German FOIS validation studies against instrumental assessment included 302, 128, and 114 patients, respectively. The relatively large size of the analyzed sample constitutes an additional strength of the present study.

Furthermore, consistent with the results reported by Hamzic et al. [12], FOIS-It has been confirmed as a valid tool to assess functional oral intake reflecting the presence of swallowing safety and efficiency analyzed during FEES examination. Functional oral intake and swallowing safety and efficiency appear to be related constructs regardless of the technique used for a swallowing examination since correlations with VFSS assessment were previously reported [9, 10].

### FOIS-It and Swallowing Safety

As regards swallowing safety, results of the present study support the convergent validity hypothesis that functional oral intake is moderately inversely associated with swallowing safety assessed with the PAS. This finding is consistent with previous research. In the study conducted by Hamzic et al. [12], the only one to assess swallowing with FEES, a strong inverse correlation between the two constructs was reported. Differences in the correlational strength might be due to the different OD etiology. Crary et al. [9] and Zhou et al. [10] found a moderate, but not significant, direct correlation between FOIS and aspiration severity. However, in these studies VFSS was used to assess aspiration and the procedure for scoring was not clearly described, making it difficult to discuss discrepancies in the findings. Regarding known-group validity, for all the consistencies tested FOIS-It distribution differed in patients with safe swallow, penetration, and aspiration, with decreased FOIS-It median values in more impaired swallowing safety groups. In addition, patients with penetration/aspiration for either liquids, or semisolids, and solids and aspiration of liquids and semisolids, compared to those without, showed a reduced functional oral intake. Changes in bolus volume and viscosity are reported as a frequently used compensatory strategy to manage impairment in swallowing safety [2, 5, 29]. Accordingly, patients with swallowing safety concerns may exhibit a reduction in functional oral intake because they are on texture-modified regimens. The absence of significant differences in FOIS-It distribution for patients with and without aspiration for solids should be interpreted with caution as it may be due to a reduced number of patients in the former group. In fact, owing to safety reasons solids were not tested in approximately one quarter of the patients since the FEES protocol establishes not to test solid consistency in patients with severe impairment in the oral control of the bolus associated with a silent aspiration of semisolids. There is a likelihood that a relevant proportion of the patients who had not been tested with solids would have shown aspiration if they had actually been tested. Thus, the patients excluded from the analyses for safety reasons presented the most impaired swallowing safety. As a result, it may be hypothesized that if the whole sample had been tested, significantly reduced FOIS-It levels would have been found in patients with an aspiration of solids compared to those without. The absence of these significant differences may thus be interpreted as a bias due to the reduced number of patients tested with solids, rather than the absence of a real difference.

### FOIS-It and Swallowing Efficiency

Rofes et al. [2] reported nutritional compromise as a health effect of impaired swallowing efficiency. Similarly, a significant reduction in functional oral intake was hypothesized when swallowing efficiency was impaired, since texture-modifications or tube-feeding may be necessary to achieve or maintain an adequate nutritional status. Conversely with the hypothesis on convergent validity of the present study, swallowing efficiency was found to be only weakly correlated with the FOIS-It, with decreasing functional oral intake corresponding to increasing amounts of residue only if located in the pyriform sinuses. However, this result cannot be discussed in the light of previous FOIS validation studies, since none of them explored the potential association between functional oral intake and swallowing efficiency. As to known-group validity, a reduced functional oral intake was found in patients with residue in the pyriform sinuses with semisolids and solids, while the presence of residue in the valleculae and in all sites with liquids did not reflect differences in functional oral intake. To discuss both convergent and known-group validity results, it is worth bearing in mind that only the presence of residue and not its management was considered for swallowing efficiency assessment in the current study. Potentially, residue management could be, instead, a variable that clarifies the relationship between functional oral intake and swallowing efficiency. Noteworthy is also the fact that swallowing safety and efficiency assessment was performed in a standard, non-ecological setting and only the worst score was considered for analysis purposes. Therefore, patients' swallowing performance during a meal could be different from what was objectified on FEES. OD was secondary to neurodegenerative disease in most of the patients in our sample. Consequently, in non-acute conditions, the level of functional oral intake could depend not only on swallowing safety and efficiency but also on other relevant factors, such as the implementation of strategies to cope with swallowing difficulties. These may include the use of clearing maneuvers, oral phase efficacy, behavioral alterations, cognitive status, caregiver assistance to meals, and environmental modifications.

### FOIS-It and Nutritional Status

In addition to the previous validation studies [9–12], FOIS-It has been established as a valid measure of functional oral intake since it reflects patients' nutritional status, both when associations with BMI (convergent validity) and comparisons between patients with and without malnutrition (known-group validity) were tested. In particular, increasing BMI corresponds to increasing functional oral intake, and malnourished patients presented a reduced functional oral intake, compared to those with normal nutritional status. In the present study, a BMI ≤ 18.5 was used as a diagnostic criterion for malnutrition [19]. Although a nutritional risk assessment with composite screening tools is recommended as a preliminary step for malnutrition diagnosis, these tools are not always used in dysphagia clinical practice, while BMI represents a quick and always available measurement, along with a thorough nutritional history.

### Study Limitations and Future Directions

The present study is not exempt from limitations. First of all, the sample is made up of patients with OD of etiological heterogeneity, mostly neurodegenerative diseases while acute conditions are less represented. As previously discussed, factors other than swallowing safety and efficiency in chronic and degenerative conditions may be reflected in the functional oral intake, thus influencing the results. Future studies will need to address samples with a homogeneous representation of both chronic and acute conditions and validate FOIS-It in subsamples which are homogeneous for diagnosis. Secondly, data are missing as regards swallowing safety and efficiency and BMI. Due to safety reasons, it was not always possible to administer all consistencies to each patient, as established in the FEES protocol, thus explaining the reason for swallowing safety and efficiency missing data. Moreover, since this study represents a secondary analysis of data collected for ongoing research projects on swallowing function, BMI missing data could not be retrieved if they had not been previously collected.

The aim of the present study was to assess known-group and convergent validity of the FOIS-It. Nevertheless, the absence of test–retest reliability analyses of the FOIS-It should be acknowledged as a limitation. It was not possible to test reliability because the present study represents a secondary analysis of data collected for ongoing research studies which did not involve a second assessment after a congruent period of time. Thus, to gain an overview of the FOIS-It psychometric properties, future studies should cover this gap.

Finally, malnutrition diagnosis was based on BMI scores only, as it is a recognized diagnostic criterion. Nevertheless, it is recommended that future research studies should consider composite nutritional screening tools and blood biomarkers to assess nutritional risk and malnutrition, respectively, as they may add information to the relationship between functional oral intake and nutritional status.

### Conclusion

FOIS-It is a valid tool, with satisfactory convergent and known-group validity, to assess functional oral intake in patients with OD from heterogeneous clinical conditions, as it reflects swallowing safety and efficiency objectified on FEES and nutritional status. FOIS-It validation presents relevant implications for clinical practice since it provides clinicians with a communal standardized language to document functional oral intake in patients with OD.

Future studies will need to expand on FOIS-It validation considering homogeneous diagnostic groups of patients, other than stroke, besides confirming the association between functional oral intake and nutritional status assessed with composite nutritional screening tools and blood biomarkers of malnutrition.
